# A membrane-bound matrix-metalloproteinase from *Nicotiana tabacum *cv. BY-2 is induced by bacterial pathogens

**DOI:** 10.1186/1471-2229-9-83

**Published:** 2009-06-29

**Authors:** Andreas Schiermeyer, Hanna Hartenstein, Manoj K Mandal, Burkhard Otte, Verena Wahner, Stefan Schillberg

**Affiliations:** 1 Fraunhofer Institute for Molecular Biology and Applied Ecology (IME), Department Plant Biotechnology, Forckenbeckstrasse 6, 52074 Aachen, Germany; 2 RWTH Aachen University, Institute for Molecular Biotechnology, Worringerweg 1, 52074 Aachen, Germany; 3 Aachen University for Applied Sciences, Campus Juelich, Ginsterweg 1, 52428 Juelich, Germany

## Abstract

**Background:**

Plant matrix metalloproteinases (MMP) are conserved proteolytic enzymes found in a wide range of monocotyledonous and dicotyledonous plant species. Acting on the plant extracellular matrix, they play crucial roles in many aspects of plant physiology including growth, development and the response to stresses such as pathogen attack.

**Results:**

We have identified the first tobacco MMP, designated NtMMP1, and have isolated the corresponding cDNA sequence from the tobacco suspension cell line BY-2. The overall domain structure of NtMMP1 is similar to known MMP sequences, although certain features suggest it may be constitutively active rather than dependent on proteolytic processing. The protein appears to be expressed in two forms with different molecular masses, both of which are enzymatically active as determined by casein zymography. Exchanging the catalytic domain of NtMMP1 with green fluorescent protein (GFP) facilitated subcellular localization by confocal laser scanning microscopy, showing the protein is normally inserted into the plasma membrane. The *NtMMP1 *gene is expressed constitutively at a low level but can be induced by exposure to bacterial pathogens.

**Conclusion:**

Our biochemical analysis of NtMMP1 together with bioinformatic data on the primary sequence indicate that NtMMP1 is a constitutively-active protease. Given its induction in response to bacterial pathogens and its localization in the plasma membrane, we propose a role in pathogen defense at the cell periphery.

## Background

Matrix metalloproteinases (MMPs) are protein-digesting enzymes that are widely distributed in the plant kingdom. Genes encoding MMPs have been cloned from several plant species including soybean, cucumber and the model legume *Medicago trunculata*, and have also been identified in sugarcane [1-6]. In *Arabidopsis thaliana*, a family of five very similar intronless MMP genes has been identified [7] encoding proteins with the same characteristic domain structure as animal MMPs [8]. This comprises an N-terminal signal peptide, a propeptide including a cysteine switch motif, and a zinc-binding region with the conserved sequence HEXGHXXGXXH followed by a methionine turn motif. Four of the *Arabidopsis *MMPs are predicted to integrate into the plasma membrane via a C-terminal hydrophobic helix, while the presence of an uncleavable signal peptide suggests the remaining family member resides in the ER lumen.

Although the natural substrates of plant MMPs are unknown, they play important roles in a variety of physiological processes including senescence [3], pathogen defense [1] and growth and development [9]. Very recently an MMP-like protein from *M. trunculata *(MtMMPL1) has been shown to be involved in the establishment of symbiotic interactions with *Sinorhizobium meliloti *[4]. In this case the protein's function might not depend on proteolytic activity since it has an amino acid substitution in a normally conserved position within the catalytic domain.

MMPs are usually expressed at low levels in a variety of tissues but are strongly induced under certain conditions. The levels of soybean *SMEP1 *and *Arabidopsis At2-MMP *mRNA in leaf tissue increase in line with the age of the plant [2,9] and *Cs1-MMP *mRNA levels in cucumber increase sharply after the onset of senescence in cotyledons and leaves [3]. *GmMMP2 *mRNA in soybean is induced by certain types of stress, including wounding, dehydration and infection with the oomycete pathogen *Phytophtora sojae *or the bacterial pathogen *Pseudomonas syringae *pv. *glycinea *[1]. *At3-MMP *mRNA in *Arabidopsis *is induced > 30-fold 30 minutes after exposure of seedlings to the *P. syringae *derived flg22 peptide [10].

Here we describe the cloning of a tobacco MMP gene from tobacco BY-2 suspension cells and functional analysis of the encoded product, NtMMP1 using zymographic assays on artificial substrates. We determined the subcellular localization of NtMMP1 using a fluorescent reporter protein, and analyzed the expression profile during normal fermentation and after challenge with bacterial pathogens. Structural and functional differences between NtMMP1 and the well-characterized vertebrate MMPs are discussed.

## Results

### Cloning the *NtMMP1 *cDNA

**Figure 1:**
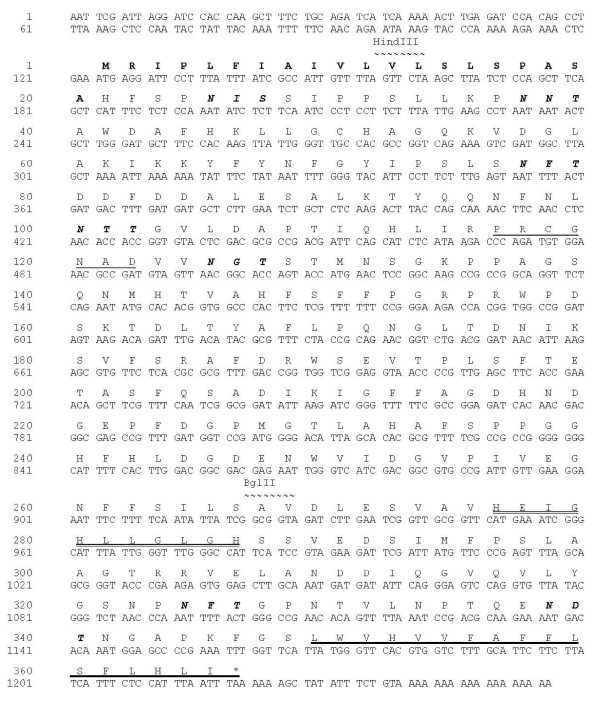
**Nucleotide and amino acid sequences of NtMMP1**. The signal peptide sequence (aa 1–20) is shown in bold. The seven potential N-glycosylation sites are shown in bold italics. The so-called cysteine switch motif is underlined, the zinc binding region within the catalytic domain is double underlined and the predicted hydrophobic transmembrane helix is underlined in bold.

Degenerate MMP primers were designed by reverse translation of the conserved zinc-binding motif in the collection of plant MMP sequences in the GenBank^® ^database. These were used to amplify MMP cDNA sequences from BY-2 cell total RNA in combination with an oligo(dT) primer. A putative partial MMP sequence was identified by sequencing several of the cloned PCR products and completed by amplification of the 5'-end of the cDNA using specific primers. The complete cDNA was 1270 bp in length and contained an open reading frame of 1098 bp encoding a 365-amino-acid MMP named NtMMP1 (Figure 1).

**Figure 2:**
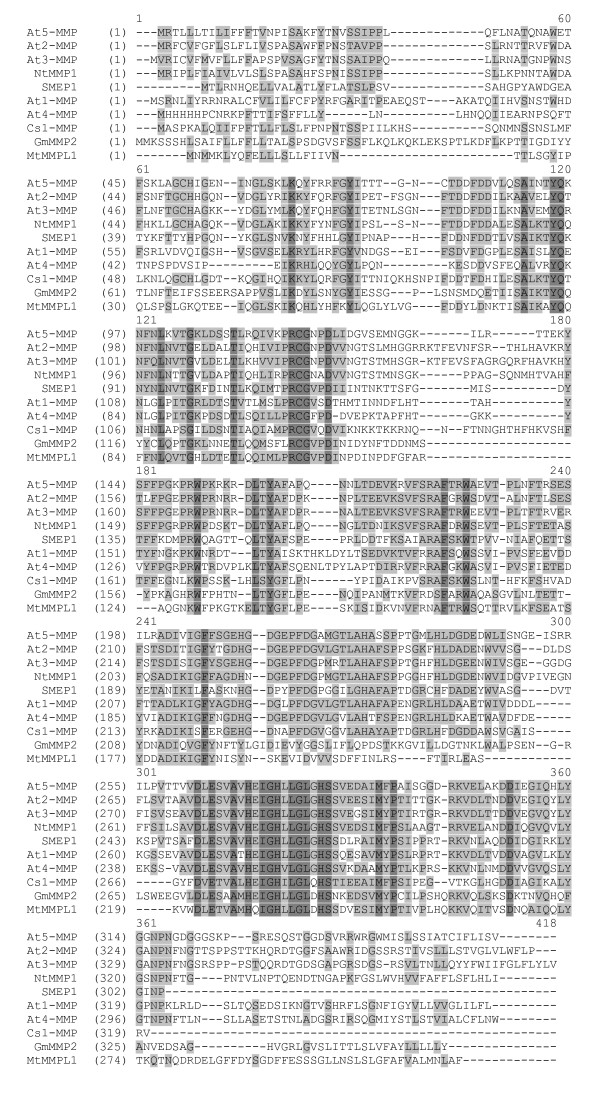
**Multiple sequence alignment of ten plant MMPs described in the literature**. The protein sequences were retrieved from GenBank with the following accession numbers: At1-MMP [GenBank: AAO42162 ], At2-MMP [GenBank: NP_177174 ], At3-MMP [GenBank: NP_173824 ], At4-MMP [GenBank: NP_182030 ], At5-MMP [GenBank: NP_176205 ], SMEP1 GenBank: P29136 ], GmMMP2 [GenBank:AAL27029 ], Cs1-MMP [GenBank: CAB76364 ], NtMMPL1 [GenBank: CAA77093 ]. Amino acid residues that are identical in all ten sequences are shown with a dark grey background, blocks of similar amino acids are shown with a light grey background.

The NtMMP1 protein sequence contained all the components found in other MMPs, including a signal peptide (aa 1–20), a potential propeptide (aa 21–145) containing a cysteine switch motif (aa 116–123), a putative peptidoglycan binding motif (aa 55–117), two zinc-binding sites (structural and catalytic), a methionine turn motif (aa 292–296), a potential transmembrane domain, and seven potential N-glycosylation sites. According to the MEROPS classification of proteases [11], NtMMP1 belongs to the M10A subfamily of plant matrixins. NtMMP1 is closely related to At2-MMP, At3-MMP and At5-MMP from *A. thaliana *with 65.6%, 65.3% and 63.8% identity at the amino acid sequence level, respectively. Figure 2 shows NtMMP1 aligned with other plant MMP sequences described in the literature.

### Subcellular localization of NtMMP1

*In silico *analysis using InterProScan [12] and PSORT [13] predicted that NtMMP1 is targeted to the secretory pathway and integrated into the plasma membrane via a C-terminal 17-amino-acid hydrophobic domain. To test this prediction, the catalytic domain of NtMMP1 was exchanged with the sequence for Emerald GFP (EmGFP), a variant of the green fluorescent protein [14]. Tobacco BY-2 cells were stably transformed with this construct and the localization of NtMMP1-GFP was analyzed by laser scanning confocal microscopy.

**Figure 3:**
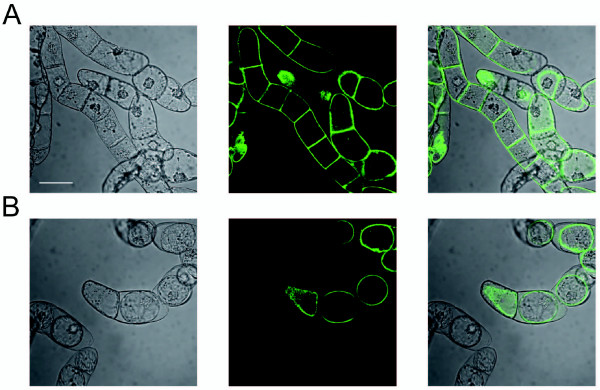
**BY-2 confocal laser scanning microscopy**. Tobacco BY-2 cells stably transformed with NtMMP1-GFP were analyzed by confocal laser scanning microscopy six days after sub-culturing. A: Untreated cells. B: Cells after treatment with 0.5 M KNO_3 _to induce plasmolysis. In each case, white light transmission is shown on the left, green fluorescence in the middle, and the overlaid images on the right. The scale bar indicates a distance of 50 μm.

By subculture day 6, confocal analysis revealed clear labeling of the plasma membranes but no significant staining in other cell compartments (Figure 3A). Additional staining of the ER was observed prior to day 6 (data not shown) indicating transit of the protein through the secretory pathway. To exclude the possibility that NtMMP1-GFP is secreted to the apoplast and not associated with the plasma membrane, cells were rinsed with 0.5 M KNO_3 _to induce plasmolysis. Under these conditions GFP staining was clearly associated with the protoplasts, whereas no GFP was detected in the surrounding cell walls, confirming membrane integration (Figure 3B).

### Transient expression of recombinant NtMMP1 and analysis of proteolytic activity

To facilitate analysis of NtMMP1 enzymatic activity, two recombinant NtMMP1 versions designated NtMMP1-apo and NtMMP1-KDEL were produced. In both variants the C-terminal hydrophobic domain was omitted to facilitate protein extraction. NtMMP1-apo contained a C-terminal His_6 _tag for purification, NtMMP1-KDEL contained the His_6 _tag followed by the ER retention sequence. The corresponding NtMMP1-apo and NtMMP1-KDEL cDNAs were inserted into the plant expression vector pTRAkt and the proteins transiently expressed in tobacco leaves. Total soluble proteins were extracted from tobacco leaves using mild detergents and recombinant NtMMP1 was purified via the C-terminal histidine tag.

**Figure 4:**
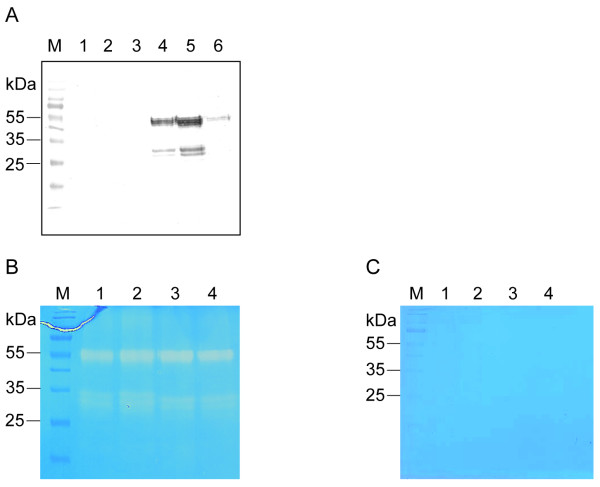
**Analysis of recombinant NtMMP1 produced transiently in tobacco leaves**. A: Immunoblot analysis of fractions from immobilized metal affinity chromatography purification of NtMMP1-apo. Equal volumes of the different fractions were separated by 12% (w/v) SDS PAGE, blotted onto nitrocellulose membranes and probed with a Penta-His antibody (Qiagen) diluted 1:5000, followed by detection with a goat anti-mouse AP-labeled Fc-specific antibody (Dianova) diluted 1:10.000 and development with NBT/BCIP. Lane 1: protein extract from wild type plants; 2: flow through fraction; 3: wash fraction; 4–6: elution fractions. B: Zymography of recombinant NtMMP1 (NtMMP1-apo and NtMMP1-KDEL). Equal amounts of NtMMP1-apo and NtMMP1-KDEL were separated by 12% (w/v) SDS PAGE containing 0.1% (w/v) casein. Lane 1: NtMMP1-KDEL with APMA treatment; 2: NtMMP1-KDEL without APMA treatment; 3: NtMMP1-apo with APMA treatment; 4: NtMMP1-apo without APMA treatment. C: Zymography in the presence of 10 mM EDTA. Samples were applied as listed in B.

Immunoblot analysis revealed that the purified recombinant NtMMP1-apo exists in two forms with apparent molecular masses of ~30 and 55 kDa (Figure 4A). The theoretical mass calculated from the amino acid sequence lacking the signal peptide is 37.7 kDa. The difference between the predicted and apparent values probably reflects glycosylation at one or more of the seven potential N-glycosylation sites. The microheterogeneity of the upper band likely reflects differences in the glycosylation pattern and represents the full-length NtMMP1 protein including the propeptide. The lower molecular weight form of NtMMP1 that appears as a double band likely represents differentially processed forms without the propeptide. Data for SMEP1 suggest that the protein could be processed in the region of amino acid residue 150 [15], which is consistent with the observed molecular mass of ~30 kDa for the low molecular weight forms of recombinant NtMMP1.

The zymography assay demonstrated that all forms of NtMMP1-apo are enzymatically active and degrade co-polymerized casein in a polyacrylamide gel, the same being true for the KDEL-tagged version of the protein (Figure 4B). Preincubation of all recombinant forms with APMA, a metallo-organic activator of metalloproteases [16], did not enhance casein degradation, indicating that recombinant NtMMP1 is already present in an active form. In contrast, enzymatic activity was efficiently blocked by the inclusion of 10 mM EDTA in the protease buffer, showing that divalent cations are required as cofactors for NtMMP1 activity (Figure 4C).

### Analysis of endogenous *NtMMP1 *expression in BY-2 cells

**Figure 5:**
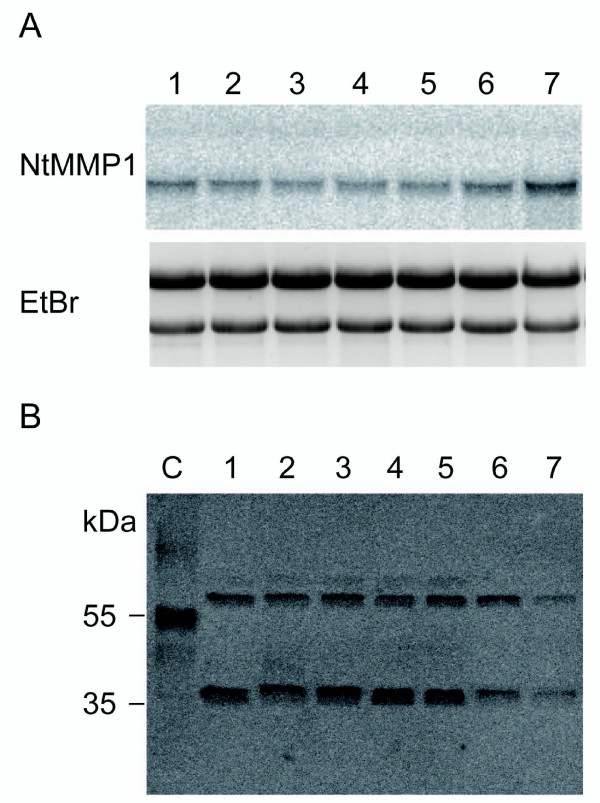
**NtMMP1 expression in wild type BY-2 suspension cells**. A: Northern blot analysis of the endogenous *NtMMP1 *mRNA during BY-2 suspension cell cultivation. Total RNA (12 μg) was loaded for each time point and the blot was hybridized with a *Bgl*II/*Hin*dIII NtMMP1 probe. The ethidium bromide bands confirm equal loading. Lane 1: day 4, 2: day 5; 3: day 6; 4: day 7; 5: day 8; 6: day 9; 7: day 10 after sub-culturing. B: Endogenous NtMMP1 protein was detected during BY-2 suspension cell cultivation by immunoblot analysis. Equal amounts of BY-2 cell extracts were separated by 12% SDS PAGE and blotted onto a nitrocellulose membrane. NtMMP1 was detected with anti-LeMMP antiserum diluted 1:2000 and a goat anti-rabbit HRP-labeled Fc-specific antibody diluted 1:5000 (Dianova) followed by the ECL procedure. C: recombinant NtMMP1-apo transiently produced in tobacco leaves as positive control. Lane 1: day 4; 2: day 5; 3: day 6; 4: day 7; 5: day 8; 6: day 9; 7: day 10 after sub-culturing.

The expression of *NtMMP1 *mRNA and NtMMP1 protein was monitored in wild type BY-2 cells between days 4 and 10 of a typical fermentation cycle. The mRNA could be detected by Northern blot at all time points although a slight increase was observed at day 10 (Figure 5A). However, the overall expression levels were quite low, perhaps providing an explanation for the absence of *NtMMP1 *sequences in the BY-2 EST database [17]. In agreement with the transient expression data, the NtMMP1 protein was represented by two forms with molecular masses of > 55 kDa and > 35 kDa (Figure 5B). In contrast to the mRNA data, the abundance of both proteins declined towards the end of the cultivation. The mobility of the larger band was slightly retarded compared to the recombinant form of NtMMP1 reflecting the presence of the hydrophobic C-terminus, which was removed from the recombinant protein.

### Induction of *NtMMP1 *by *Pseudomonas syringae*

**Figure 6:**
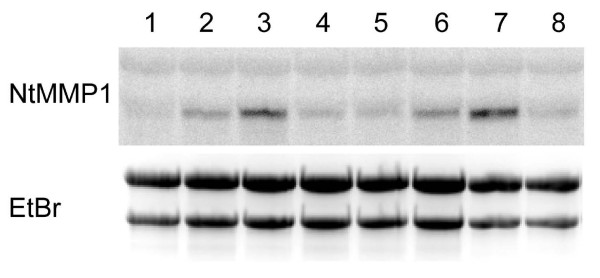
**Induction of *NtMMP1 *in wild type BY-2 suspension cells**. BY-2 cells were treated with *A*. *tumefaciens*, *P*. *syringae *pv *tomato *DC3000 or xylanase from *T. viridae*. The bacteria were grown to OD_600 _of 1.0 and diluted 1:100 in the BY-2 cell culture. Xylanase was used at a final concentration of 2 μg/ml. Total RNA was extracted at the indicated time points and 12 μg were loaded per lane. *NtMMP1 *mRNA was detected by probing with a radiolabeled *Bgl*II/*Hin*dIII *NtMMP1 *fragment. Signals were detected with a phosphorimager and quantified using the AIDA software. The ethidium bromide bands confirm equal loading. Lane 1: 30 min untreated cells; lane 2: 30 min exposure to *A. tumefaciens*; lane 3: 30 min exposure to *P. syringae*; lane 4: 30 min exposure to xylanase; lane 5: 1 h untreated cells; lane 6: 1 h exposure to *A. tumefaciens*; lane 7: 1 h exposure to *P. syringae*; lane 8: 1 h exposure to xylanase.

To determine whether NtMMP1 can be induced by pathogens like other plant MMPs, BY-2 cells were incubated with either *Agrobacterium tumefaciens*, *Pseudomonas syringae *pv *tomato *or xylanase from *Trichoderma viridae *[18]. Total RNA was isolated after 30 min and 1 h and Northern blots were carried out using *NtMMP1 *as the probe (Figure 6). While *NtMMP1 *mRNA levels are induced after treatment with *P. syringae *and *A. tumefaciens*, the xylanase treatment had no effect on *NtMMP1 *mRNA levels indicating a lack of responsiveness toward fungal elicitors.

The induction level of *NtMMP1 *mRNA after one hour of incubation with either *P. syringae *or *A. tumefaciens *were calculated from three independent biological replicates using the AIDA software. For the *Agrobacterium *treatment the calculated induction factor is 2.4 (SD = 0.9) and for the *Pseudomonas *treatment 5.1 (SD = 1.1).

## Discussion

We have cloned a cDNA encoding the matrix metalloproteinase NtMMP1 from tobacco BY-2 cells, which possess all the expected features of a MMP including the cysteine switch, and the zinc-binding region and methionine turn motif in the catalytic domain. Although the overall structure is very similar to other MMPs, NtMMP1 also has some novel features, including the substitution of alanine for the second proline residue normally found within the cysteine switch consensus sequence PRCXXPD [8]. Since proline residues have a profound impact on protein structure, substitution with the non-polar amino acid alanine may lead to the inactivation of the cysteine switch by preventing the free cysteine residue coordinating the zinc ion within the catalytic domain and maintaining the latency of the proenzyme. The sensitivity of this motif towards amino acid replacements has been shown for the human MMP-26 where an arginine to histidine exchange within this domain inactivates the cysteine switch [19]. This amino acid substitution leads to structural changes within the prodomain and hence to an alternative activation mechanism that is independent of the cysteine switch motif.

Another key feature is that NtMMP1 contains a second cysteine residue (Cys 50) in the N-terminal portion of the protein. According to the Scratch protein predictor server [20] this residue is predicted to form a disulfide bridge with Cys 118 in the cysteine switch motif. Therefore, it is unlikely that NtMMP1 is regulated by the cysteine switch mechanism that has been proposed for human MMP molecules [21]. The closest homologs to NtMMP1 are At2-MMP, At3-MMP, and At5-MMP from *A. thaliana *which also contain one (At2-MMP and At3-MMP) or two additional cysteine residues (At5-MMP). The additional cysteine residues in these MMPs are also predicted to form disulfide bridges with the cysteine residue from the switch motif, possibly representing constitutively active forms of the enzyme. Like NtMMP1, they have a C-terminal hydrophobic domain and are believed to reside in the plasma membrane [7].

The above data suggest that NtMMP1 does not require proteolytic cleavage for activation, a hypothesis supported by the finding that APMA treatment has no effect on NtMMP1 activity. Although it is well established that zymogens are activated stepwise during zymography [22], APMA treatment is accompanied by a decrease in molecular mass due to autoproteolytic processing [23]. However, we observed no shift to a lower molecular mass in the NtMMP1 zymogram assay (Figure 4B). Furthermore both recombinant forms NtMMP1-apo and NtMMP1-KDEL show the same activity although they are expected to have different subcellular localizations. While NtMMP1-KDEL is expected to reside exclusively in the ER due to the C-terminal KDEL sequence, NtMMP1apo can follow the entire secretory pathway until it is finally secreted to the apoplast. Therefore NtMMP1 seems to gain enzymatic activity immediately after synthesis in the ER. Since no endogenous MMP inhibitor proteins like the tissue inhibitors of metalloproteases (TIMPs) in animals have been identified thus far in plants, it is likely that NtMMP1 is constitutively active.

NtMMP1 is expressed constitutively but at a low level during BY-2 cell cultivation (Figure 5). The low expression level is reflected by the absence of NtMMP1-related sequences in an EST library of BY-2 cells containing more than 9200 sequences [17]. *NtMMP1 *mRNA is induced within 30 min after the treatment of BY-2 cells with *P. syringae *and to a lesser extent by *A. tumefaciens *(Figure 6). Other MMP genes induced by pathogenic bacteria include soybean *Gm*MMP2, which is induced after treatment with compatible and incompatible *P. syringae *pathovars [1], and *Arabidopsis *At3-MMP, which is rapidly induced after treatment of *Arabidopsis *seedlings with a 22-amino-acid peptide (flg22) derived from *P. syringae *flagellin [10]. The normal substrates for NtMMP1 are unknown, so it may act directly against invading bacteria or may help to generate signaling molecules that trigger further defense responses of the plant cell. Given the constitutive expression and activity of NtMMP1, it might be an integral part of the plant's surveillance system for pathogens or other stress signals.

The N-terminal portion of NtMMP1 (aa 55–117) is predicted to form a peptidoglycan-binding motif comprising three alpha helices, a structure initially described for the *Streptomyces albus *Zn^2+ ^G peptidase [24]. According to the Pfam protein families database [25] many matrixins contain an N-terminal peptidoglycan-binding like motif (PF01471). Whether this domain binds to bacterial pathogen-associated molecular patterns (PAMPs) such as peptidoglycan [26] and flagellin [10] remains to be determined. The plant cell usually recognizes specific peptide fragments from PAMPs rather than the full length proteins [27,28]. In the case of flagellin, a peptide fragment from the DO domain is recognized by the corresponding plant surface receptor [29]. Yet this domain, and hence the flg22 peptide that binds to the plant FLS2 receptor, is hidden inside the intact bacterial flagellum [30]. It is therefore tempting to speculate that plasma membrane-bound proteases such as NtMMP1 recognize PAMPs and process them to generate specific peptides that subsequently bind to their corresponding transmembrane receptors of the nucleotide-binding site/leucine-rich repeat (NBS-LRR), receptor-like kinase (RLK) or receptor-like protein (RLP) classes [31]. Although *NtMMP1 *did not respond to the fungal elicitor xylanase (Figure 6) MMP induction has been shown in soybean for *Gm*MMP2 treated with the oomycete *P. sojae *and in tomato for LeMMP1 treated with the fungal elicitor fusicoccin [32]. Therefore also certain PAMPs from fungal origin are able to induce MMP expression. In future work we will aim to determine the natural substrate(s) of NtMMP1 and its potential role in PAMP recognition and processing. However, the induction of NtMMP1 by bacterial pathogens indicates its involvement in pathogen recognition and defense responses and therefore contributes to our understanding of pathogen-host interactions.

## Conclusion

The matrix metalloproteinase NtMMP1 is localized in the plasma membrane of tobacco BY-2 cells. Our biochemical data indicate that the enzyme is constitutively active, and this is supported by bioinformatic analysis of the primary sequence. The low basal level of *NtMMP1 *expression increases immediately after the exposure of tobacco BY-2 cells to bacterial pathogens. Given the low-level constitutive activity of the protein, its induction in response to bacterial pathogens and its localization at the cell surface, we propose that NtMMP1 plays a role in pathogen recognition and defense at the cell periphery.

## Methods

### Gene cloning

Degenerate primers were designed according to the CODEHOP procedure [33] based on the known MMP protein sequences from *Arabidopsis thaliana*, soybean, rice, cucumber and *Medicago trunculata *[GenBank: NP_177174 , GenBank: NP_176205 , GenBank: NP_173824 , GenBank: O65340 , GenBank: NP_182030 , GenBank: AAM62476 , GenBank: O48680 , GenBank: AAO42162 , GenBank: P29136 , GenBank: 1905425A , GenBank: AAL27029 , GenBank: AAK55464 , GenBank: AAK55462 , GenBank: AAK55459 , GenBank: CAB76364 , GenBank: CAA77093 ]. Total RNA was prepared from logarithmically growing *Nicotiana tabacum *cv Bright Yellow 2 (BY-2) cells using the RNeasy Plant Mini Kit (Qiagen, Hilden, Germany) and a cDNA was synthesized using the MM3 primer (5'-CTC GAG GAT CCG CGG CCG C(T)_18_-3') and the Superscript first strand cDNA synthesis system (Invitrogen, Karlsruhe, Germany). MMP-related sequences from BY-2 cDNA were amplified with the primer pair Metallo-1 (5'-GAT CTG GAA TCT GTT GCT GTT CAY GAR ATH GGN C-3') and MM3, in a 50-μl reaction volume using the Expand High Fidelity PCR System (Roche, Mannheim, Germany). The program comprised 5 min at 95°C followed by 35 cycles of denaturation at 95°C for 30 s, annealing at 53°C for 30 s and extension at 72°C for 30 s. PCR products were gel purified and cloned in the pCR2.1 vector using the TOPO Cloning Kit (Invitrogen). Insert sequences were verified using the BigDye Sequencing Kit (Applied Biosystems, Darmstadt, Germany).

To clone the missing 5' portion of the *MMP *sequence, the adapter ASLinker (5'-PO_4_-CTG CAG AAA GCT TGG TGG ATC CTA-NH_2_-3') was ligated to single stranded cDNA as described [34]. Using the complementary primer AS04 (5'-TAG GAT CCA CCA AGC TTT CTG CAG-3') and the MMP-specific primer MMPRace1 (5'-GGG TTA GAC CCG TAT AAC ACC TGG AC-3') the 5' end of the cDNA was amplified using the PCR procedure described above and the following program: 5 min at 95°C followed by 35 cycles of denaturation at 95°C for 1 min, annealing at 50–70°C for 30 s and extension at 72°C for 1 min. PCR products were subcloned and sequenced as described above. The final *NtMMP1 *full-length cDNA sequence was deposited in GenBank^® ^[GenBank: DQ508374].

### Transient expression of recombinant NtMMP1

To produce recombinant NtMMP1 for functional analysis, the 5' and 3' cDNA sequences were amplified, joined in-frame by SOE-PCR [35] and inserted into the plant expression vector pTRAkt [36]. To facilitate extraction of the recombinant protein, the hydrophobic C-terminal transmembrane domain was omitted. Two constructs were generated, one with a C-terminal His_6 _tag alone (NtMMP1-apo) and another with a C-terminal His_6 _tag followed by a SEKDEL motif for ER retention (NtMMP1-KDEL). The 5' portion of NtMMP1 was amplified using primers NtMMP1-Nterm_for (5'-*CCA TGG *AAA TGA GGA TTC CTT TAT TTA TCG CC-3') and NtMMP1-Nterm_rev (5'-CCA CTC TTC GGG TAC CCG CTG C-3'). The 3' portion was similarly amplified using primers NtMMP1-Cterm_for (5'-AGC AGC GGG TAC CCG AAG AGT GGA GC-3') and either NtMMP1-Cterm-apo_rev (5'-*TCT AGA *CTA GTG ATG GTG ATG GTG ATG ACC AAA TTT CGG GGC TCC ATT TGT GTC-3') or NtMMP1-Cterm-KDEL_rev (5'-*GCG GCC GC*A CCA AAT TTC GGG GCT CC-3'). Introduced restriction sites are shown in italic. The amplified partial cDNAs were joined by SOE-PCR and inserted into pTRAkt using the *Nco*I and *Xba*I sites for the NtMMP1-apo construct or the *Nco*I and *Not*I sites for the NtMMP-KDEL construct.

Both vectors were introduced into *A. tumefaciens *GV3101::pMP90RK by electroporation [37]. The recombinant proteins were expressed transiently in detached leaves of *N. tabacum *cv. Petite Havana SR1 by vacuum infiltration [38] and partially purified via their His_6 _tags by immobilized metal-affinity chromatography (IMAC) as described previously [39].

### GFP fusions

To analyze the cellular localization of recombinant NtMMP1 by fluorescence microscopy, the peptidase domain was replaced with the cDNA encoding Emerald GFP (EmGFP, Invitrogen). The 5' end of the *NtMMP1 *cDNA was amplified with the primer pair NtMMP1-Nterm_for (5'-*CCA TGG *AAA TGA GGA TTC CTT TAT TTA TCG CC-3') and NtMMP-Nterm+GFP_rev (5'-CTC GCC CTT GCT CAC CAT ATT CTG AGA ACC TGC CGG CG-3'), EmGFP was amplified with the primer pair GFP_for (5'-CGC CGG CAG GTT CTC AGA ATA TGG TGA GCA AGG GCG AG-3') and GFP_rev (5'-GGC CCA GTA AAA TTT GGG TTA GAC TTG TAC AGC TCG TCC ATG CCG-3'), and the 3' end of the *NtMMP1 *cDNA was amplified with the primer pair NtMMP1-Cterm+GPF_for (5'-CGG CAT GGA CGA GCT GTA CAA GTC TAA CCC AAA TTT TAC TGG G-3') and NtMMP-Cterm_rev (5'-*TCT AGA *TTT AAA TTA AAT GGA GAA ATG ATA AG-3'). Introduced restriction sites are shown in italic. The three fragments were joined by SOE-PCR, reamplified, and cloned in the plant expression vector pTRAkt using the *Nco*I and *Xba*I restriction sites.

### Plant cell culture, transformation, and treatments

*N. tabacum *cv BY-2 cells [40] were maintained in MSMO medium (Sigma, Taufkirchen, Germany) supplemented with 0.15 μg/ml thiamin, 0.02 μg/ml KH_2_PO_4 _and 3% (w/v) sucrose (pH 5.6). The cells were passed each week into fresh culture medium using a 2% (v/v) inoculum for wild type and a 5% (v/v) inoculum for transgenic cells. The cells were incubated in an orbital shaker (New Brunswick Scientific, Edison, NJ, USA) at 180 rpm, 26°C in darkness.

Transgenic BY-2 cells were produced by co-cultivation with *A. tumefaciens *as described [41]. The recombinant pTRAkt vectors were transformed into *A. tumefaciens *GV3101::pMP90RK [42] by electroporation using a multiporator (Eppendorf, Hamburg, Germany).

*A. tumefaciens *was grown in YEB medium [43]. *P. syringae *pv. *tomato *DC3000 was cultivated in KingsB medium [44]. For the treatment of tobacco BY-2 cells, the bacteria were grown to an OD_600 _of 1.0 and diluted 1:100 with the BY-2 culture. Xylanase from *T. viridae *(Sigma) was used at a final concentration of 2 μg/ml.

### Plant cell confocal imaging

Wild type and transgenic BY-2 cells were imaged using a Leica TCS-SP spectral confocal microscope equipped with an argon ion laser using a 40 × oil immersion Plan-Apo objective (Leica, Wetzlar, Germany). EmGFP was excited with the 488 nm wavelength argon laser line and confocal images were taken at a 500–570 nm emission setting using Leica TCS-SP software. Image overlays were generated using Adobe Photoshop CS2 software.

### Northern blot

Total RNA was extracted from tobacco BY-2 suspension cells using the RNeasy Plant Mini Kit (Qiagen), and 12 μg were loaded onto denaturing formaldehyde agarose gels followed by capillary blotting onto nylon membranes (Hybond N^+^, GE Healthcare, Freiburg, Germany). The membranes were probed with a 765-bp *Bgl*II/*Hin*dIII fragment of the *NtMMP1 *cDNA radiolabeled with [α^32^]P-dATP (GE Healthcare) using the DecaLabel DNA labeling kit (Fermentas, St. Leon-Rot, Germany) according to the manufacturer's instructions. After prehybridization (50% (v/v) formamide, 10% (w/v) dextran sulfate, 1% (w/v) SDS, 1 M NaCl) for three hours at 42°C, the denatured probe was added to the prehybridization solution with 100 μg salmon sperm carrier DNA and hybridization was carried out at 42°C overnight. The membranes were washed twice for 30 min in 2× SSC containing 0.1% (w/v) SDS at 65°C. The signals were visualized by exposing the membranes on a phosphorimager plate overnight. The plates were read with a phosphorimager (FLA-2000, Fujifilm, Tokyo, Japan) and the images were processed using AIDA software (Raytest, Straubenhardt, Germany).

### Zymography

Protease activity was visualized by in-gel assays using casein as a substrate [45]. The substrate was co-polymerized with the acrylamide at a final concentration of 0.1% (w/v). SDS-PAGE was carried out on a 12% (w/v) gel at a constant current of 20 mA (MiniProteanII, Biorad, Munich, Germany). The samples were neither reduced nor boiled prior to loading and electrophoresis was carried out in an ice bath. After electrophoresis the SDS was removed by washing the gel twice for 15 min in 2.5% (v/v) Triton X-100 followed by two further 15-min washes in protease assay buffer (50 mM Tris, 5 mM CaCl_2_, 100 μM ZnCl_2_, pH 7.6). The gels were incubated overnight in the protease assay buffer then stained with Coomassie brilliant blue. Proteolytic activities were revealed after destaining as clear bands on a blue background.

APMA treatment was done with a final concentration of 10 mM for 2 h at 37°C as described [46].

### Immunoblot analysis

Protein samples from BY-2 cells were prepared as described [39] and separated by SDS-PAGE. The proteins were transferred onto nitrocellulose membrane by semi-dry electroblotting using a Trans-blot SD device (Biorad) and a standard transfer buffer (25 mM Tris, 192 mM, 20% (v/v) methanol, as described [47] at a constant current of 2.5 mA/cm^2 ^for 40 min. Nonspecific binding sites were blocked with 5% (w/v) skimmed milk in PBST at 4°C overnight. The membrane was washed once with PBST and NtMMP1 was detected with a rabbit anti-LeMMP antiserum raised against LeMMP from tomato (*Solanum lycopersicum*) at a dilution of 1:2000 in PBST for 1 h at room temperature. The antiserum was kindly provided by A. Schaller (University of Hohenheim, Germany). Membranes were washed three times for 5 min in PBST and incubated with a HRP-conjugated secondary goat-anti-rabbit IgG Fc_γ _antibody (Dianova, Hamburg, Germany) diluted 1:5000 in PBST. The membranes were washed three times with PBST, once with PBS and then developed with the ECL reagent (GE Healthcare). Images were acquired using the LAS 3000 cooled CCD camera device (Fujifilm).

## Abbreviations

APMA: 4-aminophenylmercuric acid; ECL: enhanced chemiluminescence; EST: expressed sequence tags; GFP: green fluorescent protein; IMAC: immobilized metal affinity chromatography; MMP: matrix metalloproteinase; MSMO: Murahige & Skoog medium with minimal organics; PAGE: polyacrylamide gel electrophoresis; SDS: sodium dodecylsulfate; SOE-PCR: splicing by overlap extension polymerase chain reaction.

## Authors' contributions

AS conceived of the study, cloned the *NtMMP1 *cDNA from BY-2 cells and participated in drafting the manuscript. HH and MKM cloned constructs for transient expression and characterized the recombinant enzyme. BO analyzed the expression of native NtMMP1 in BY-2 cells. VW cloned GFP constructs and analyzed the subcelluar localization together with BO. SS participated in the experiment design, interpretation of the data and drafting of the manuscript. All authors have read and approved the manuscript.
